# Simultaneous Determination of Aflatoxin B1 and Ochratoxin A in Cereals by a Novel Electrochemical Aptasensor Using Metal–Organic Framework as Signal Carrier

**DOI:** 10.3390/foods13142177

**Published:** 2024-07-10

**Authors:** Yiwei Xu, Xupeng Jia, Sennan Yang, Mengrui Cao, Baoshan He, Wenjie Ren, Zhiguang Suo

**Affiliations:** 1 School of Food Science and Technology, National Engineering Research Center of Wheat and Corn Further Processing, Henan University of Technology, Zhengzhou 450001, China; jiaxupeng2000@163.com (X.J.); caomengrui1213@126.com (M.C.); wjren0818@163.com (W.R.); zg_suo@163.com (Z.S.); 2 Henan Institute of Food and Salt Industry Inspection Technology, Zhengzhou 450003, China

**Keywords:** multiplex detection, aflatoxin B1, ochratoxin A, metal–organic framework, electrochemical aptasensor, cereals

## Abstract

A novel electrochemical aptasensor was prepared for the simultaneous determination of aflatoxin B1 (AFB1) and ochratoxin A (OTA). Composites of Au nanoparticles and polyethyleneimine-reduced graphene oxide (AuNPs/PEI-RGO) with good electrical conductivity and high specific surface area were employed as the supporting substrate, demonstrating the ability to provide more binding sites for aptamers and accelerate the electron transfer. Aptamers were immobilized on a AuNPs/PEI-RGO surface to specifically recognize AFB1 and OTA. A metal–organic framework of UiO-66-NH_2_ served as the signal carrier to load metal ions of Cu^2+^ and Pb^2+^, which facilitated the generation of independent current peaks and effectively improved the electrochemical signals. The prepared aptasensor exhibited sensitive current responses for AFB1 and OTA with a linear range of 0.01 to 1000 ng/mL, with detection limits of 6.2 ng/L for AFB1 and 3.7 ng/L for OTA, respectively. The aptasensor was applied to detect AFB1 and OTA in cereal samples, achieving results comparable with HPLC-MS, with recovery results from 92.5% to 104.1%. With these merits of high sensitivity and good selectivity and stability, the prepared aptasensor proved to be a powerful tool for evaluating contaminated cereals.

## 1. Introduction

Cereals are a staple food for people in most countries because of their rich nutrients. They are easily contaminated by spoilage fungi in the field and after harvest. In growth and reproduction, fungi produce a variety of metabolites. Mycotoxin is a kind of secondary metabolite produced by fungi, which is known to be toxic to humans. As previously reported, more than a quarter of food crops in the world are contaminated with mycotoxins [1]. In general, mycotoxins are stable and not easily destroyed by food processing methods [2]. Thus, they may remain in finished foods and threaten human health. Aflatoxins and ochratoxins are two kinds of common and dangerous mycotoxins in cereals. In particular, aflatoxin B1 (AFB1) is classified as a Group 1 carcinogen and ochratoxin A (OTA) as a Group 2B carcinogen by the International Agency for Research on Cancer. In many cases, AFB1 and OTA co-exist in cereals [3]. Mycotoxin contamination is strictly controlled, and maximum limits have been established worldwide. The detection of mycotoxins is an effective way to prevent mycotoxins from entering the food chain. The typical methods for mycotoxins detection are based on chromatographic and immunological techniques. Chromatographic methods included high-performance liquid chromatography (HPLC), liquid chromatography tandem mass spectrometry (MS), and gas chromatography tandem mass spectrometry [4]. These chromatographic methods are sensitive and accurate in detection, though they rely on large equipment, require complicated operation, and involve tedious pretreatment processes [5]. Immunochemical analysis methods mainly include enzyme-linked immunosorbent assay and lateral flow devices, providing a convenient route for mycotoxin screening [6]. Nevertheless, antibodies and enzymes in immunochemical analysis are easily denatured because of the fragile nature of protein, leading to unavoidable differences among batches and unstable results in immunochemical analysis [7]. There is still a need to develop rapid, sensitive, and practical methods for AFB1 and OTA detection.

Recently, electrochemical aptasensors have attracted significant attention because of their rapid response and high selectivity. Electrochemical aptasensors utilize aptamers as recognition elements to capture analytes and then generate measurable electrical signals for quantitative detection [8]. Aptamers, as a kind of single-stranded nucleic acid, can fold into various structures (hairpin, bulge loop, G-quartet, pseudoknot, etc.) by molecular interactions such as hydrophobic interaction, hydrogen bond, and van der Waals force. Their 3D structure endows the aptamer with the ability to specifically bind to target analytes [9]. In comparison to antibodies, aptamers possess some unique advantages including a high association constant, long-term stability, easy preparation, and convenient modification [10]. With the development of in vitro selection techniques, a series of aptamers for various mycotoxins have been selected through the systematic evolution of ligands by the exponential enrichment procedure [11]. The popularity of aptamers has promoted the rapid development of an aptamer-based electrochemical sensor for AFB1 and OTA detection.

Signal producers are critical to improve the sensitivity of electrochemical sensors. Various signal producers have been used in electrochemical sensors, such as enzymes, dyes, and metal ions. Owing to their excellent electrical activity, metal ions can be directly measured by electrochemical techniques, thus avoiding cumbersome operations [12]. Because different metal elements have their own unique redox potential [13], they are able to produce independent current peaks in electrochemical voltammetry, providing a feasible solution for multi-analysis. However, each individual metal ion only produces relatively weak electrochemical signals. In order to improve the current signal, an ideal carrier is still required to load plenty of metal ions. A metal–organic framework (MOF) is a kind of crystalline material constructed by assembling metal ions with multidentate organic ligands via coordination bonds. Owing to their high porosity and excellent adsorption capacity, MOFs can absorb an enormous amount of metal ions. Hence, the response signals are improved using MOFs as the signal carrier for loading metal ions, as well as the sensitivity of electrochemical sensor. UiO-66-NH_2_ is a kind of MOF that is synthesized using Zr^4+^ as a node and 2-aminoterephthalic acid as the linker. Amino groups play an important role in the binding and stabilization of metal ions (Cu^2+^, Pb^2+^, etc.) in MOFs [14,15]. Because UiO-66-NH_2_ has abundant amino groups, it can chelate an enormous amount of Cu^2+^ and Pb^2+^. Moreover, the abundant grafting groups of UiO-66-NH_2_ provide many active sites for the immobilization of biological recognition elements. With these unique features, UiO-66-NH_2_ is highly valuable in the development of novel electrochemical probes for multiple target detection.

In this work, an electrochemical aptasensor using UiO-66-NH_2_ as the signal carrier was prepared for the simultaneous determination of AFB1 and OTA. In order to immobilize aptamer molecules, the composites of Au nanoparticles and polyethyleneimine-reduced graphene oxide (AuNPs/PEI-RGO) were synthesized and deposited on the electrode surface as a supporting substrate. AuNPs/PEI-RGO composites demonstrated good electrical conductivity and high specific surface area, providing more anchor sites for aptamer molecules. The aptamer molecules were immobilized on AuNPs by Au-S bond to specifically recognize AFB1 and OTA. UiO-66-NH_2_ was synthesized and used as the signal carrier to load Cu^2+^ and Pb^2+^. Metal ions of Cu^2+^ and Pb^2+^ have different redox potentials, which could produce well-defined and separated current peaks [16,17]. These two independent peaks could reflect AFB1 and OTA concentrations, respectively. Therefore, AFB1 and OTA were detected simultaneously using UiO-66-NH_2_ as the support for Cu^2+^ and Pb^2+^. In addition, the abundant metal ions on UiO-66-NH_2_ contributed to enhancing the electrochemical signals and improving the sensitivity of the electrochemical sensor.

## 2. Materials and Methods

### 2.1. Reagents and Materials

Trisodium citrate dihydrate, tetrachloroauric(Ⅲ) acid trihydrate (HAuCl_4_·3H_2_O), N,N-Dimethylformamide (DMF), lead chloride (PbCl_2_), zirconium(IV) chloride (ZrCl_4_), 2-aminoterephthalic acid (NH_2_-BDC), 6-Mercapto-1-hexanol (MCH), potassium ferricyanide (K_3_[Fe(CN)_6_]), potassium ferrocyanide trihydrate (K_4_[Fe(CN)_6_]·3H_2_O), potassium chloride (KCl), acetic acid, ethanol, copper dinitrate (Cu(NO_3_)_2_), sodium phosphate dibasic (Na_2_HPO_4_), monosodium phosphate (NaH_2_PO_4_), and glutaraldehyde were purchased from Shanghai Macklin Biochemical Technology Co., Ltd. (Shanghai, China). Polyethyleneimine (PEI) was purchased from Shanghai Aladdin Biochemical Technology Co., Ltd. (Shanghai, China). AFB1, OTA, aflatoxin G1 (AFG1), fumonisin B1 (FB1), ochratoxin B (OTB), and zearalenone (ZEN) were obtained from Alta Scientific Co., Ltd. (Tianjin, China). Graphene oxide (GO) was obtained from Jiangsu Xianfeng nanomaterials Technology Co., Ltd. (Nanjing, China).

The DNA fragments were specially customized from Sangon Biotechnology Co., Ltd. (Shanghai, China). with HPLC purification. The aptamers of AFB1 and OTA were selected according to previous studies in the literature [18]. The sequences of DNA fragments were as follows.

AFB1 aptamer (Apt1): 5′-HS-(CH_2_)_6_-GTT GGG CAC GTG TTG TCT CTC TGT GTC TCG TGC CCT TCG CTA GGC CCA CA-3′.

cDNA of AFB1 aptamer (cDNA1): 5′-NH_2_-(CH_2_)_6_-TGT GGG CCT AGC GAA GGG C-3′.

OTA aptamer (Apt2): 5′-HS-(CH_2_)_6_-GAT CGG GTG TGG GTG GCG TAA AGG GAG CAT CGG ACA-3′.

cDNA of OTA aptamer (cDNA2): 5′-NH_2_-(CH_2_)_6_-TGT CCG ATG CTC-3′.

### 2.2. Apparatus

The transmission electron microscope (TEM) image was taken from a Talos F200X G2 microscope (Thermo Fisher Scientific, Waltham, MA, USA). The X-ray photoelectron spectroscopy (XPS) data were obtained from a K-Alpha X (Thermo Fisher Scientific, Waltham, MA, USA). The X-ray diffraction (XRD) spectrum was collected from a SmartLab SE diffractometer (Rigaku, Tokyo, Japan). The Fourier transform-infrared spectroscopy (FT-IR) information was acquired by a Nicolet iS20 infrared spectrometer (Thermo Fisher Scientific, Waltham, MA, USA). The content of AFB1 and OTA in cereal samples was analyzed by a Xevo TQ-S HPLC-MS system (Waters, Milford, MA, USA). The electrochemical experiments were conducted by a 660E electrochemical workstation (Shanghai CHI, Shanghai, China) with a three-electrode system: glassy carbon electrode (GCE) as working electrode, platinum wire electrode as counter electrode, and Ag/AgCl electrode as reference electrode.

### 2.3. Preparation of Signal Probes

MOF nanomaterial of UiO-66-NH_2_ was synthesized by a typical solvothermal method [19]. Firstly, 0.18 g of ZrCl_4_ was added in 40 mL DMF solvent and dissolved by ultrasound for 30 min. Secondly, 0.14 g of NH_2_-BDC was added in the above mixture and treated by ultrasound for 30 min. Subsequently, 4.8 mL of acetic acid was mixed with the above mixture, and then reacted in an oven at 120 °C for 24 h. After that, the product was collected by centrifugation (5000 rpm for 10 min) and then washed by DMF and ethanol three times. Finally, the product was dried at 70 °C to obtained UiO-66-NH_2_.

The signal probe of Cu^2+^@UiO-66-NH_2_-cDNA1 was prepared as follows. An amount of 2 mg of UiO-66-NH_2_ was added in 2 mL of 10 mmol/L Cu^2+^ solution and mixed by vibration for 24 h. The product of Cu^2+^@UiO-66-NH_2_ was collected by centrifugation and washed with 0.1 mol/L PBS (pH = 7.0) three times. After that, the product of Cu^2+^@UiO-66-NH_2_ was dispersed in 2 mL PBS containing 2.5% glutaraldehyde and stirred gently for 2 h. After stirring, the product was washed and dispersed in 2 mL PBS. Next, 30 μL of 0.01 mmol/L cDNA1 was added to the above dispersion, which was incubated for 1 h. After incubation, the obtained Cu^2+^@UiO-66-NH_2_-cDNA1 was washed by PBS, dispersed in 2 mL PBS, and stored at 4 °C for further use. The signal probe of Pb^2+^@UiO-66-NH_2_-cDNA2 was prepared with a similar procedure to that of Cu^2+^@UiO-66-NH_2_-cDNA1.

### 2.4. Preparation of the Electrochemical Aptasensor

AuNPs were synthesized by a common chemical reduction method [20]. Next, 1 mL of 1% HAuCl_4_ was added to 99 mL of ultrapure water. The above solution was stirred and heated on a magnetic stirrer. After boiling, 2 mL of 1% trisodium citrate solution was added quickly. When the solution color was burgundy, the solution was heated for another 10 min. After cooling down, the AuNP solution was stored at 4 °C for future use.

The composites of AuNPs/PEI-RGO were prepared to use as the substrate of the electrochemical sensor. PEI was a cationic polymer with reductive amino groups. It was covalently bonded to the GO surface through amino and carboxy. The agglomeration of graphene sheets were effectively inhibited. Furthermore, the abundant amino groups provided plenty of active sites for the subsequent attachment of AuNPs [21]. An amount of 10 mL of 1 mg/mL GO was mixed with 1 mL of 3% PEI. The mixture was treated by ultrasound for 30 min, and then heated at 135 °C for 3 h. The product of PEI-RGO was collected by centrifugation, washed with ultrapure water, and then dispersed in 4 mL ultrapure water. The above PEI-RGO dispersion was mixed with 4 mL of the prepared AuNPs solution, and then oscillated for 24 h. The product of AuNPs/PEI-RGO was collected by centrifugation, washed with ultrapure water, and then dispersed in 4 mL ultrapure water and stored at 4 °C for future use.

Prior to modification, the GCE was polished with alumina slurry and cleaned by ultrasonication. Because 5 microliters of each material could completely cover the GCE surface, the drop coating volume was 5 microliters in the study. After cleaning, 5 μL of AuNPs/PEI-RGO was dropped on the GCE surface and dried at 37 °C. The aptamer stock solution (100 μmol/L) was activated with 10 mmol/L TCEP for 1 h to cleave the disulfide bond. The activated aptamer was diluted to 1 μmol/L by Tris-HCl buffer (10 mmol/L, pH = 7.4). Next, 5 μL of freshly activated aptamer (Apt1:Apt2 = 1:1) was dropped on AuNPs/PEI-RGO/GCE surface and stored at 4 °C for 2 h. The aptamer molecules were immobilized on AuNPs/PEI-RGO/GCE by the conjunction of Au-S bond. To block the nonspecific binding sites, the electrode was immersed in 1 mmol/L MCH at 4 °C for 1 h. Next, 5 μL of the mixture of signal probes (Cu^2+^@UiO-66-NH_2_-cDNA1:Pb^2+^@UiO-66-NH_2_-cDNA2 = 1:1) was dropped on the electrode surface and incubated at 37 °C for 50 min. During incubation, the signal probes of Cu^2+^@UiO-66-NH_2_-cDNA1 and Pb^2+^@UiO-66-NH_2_-cDNA2 were immobilized on the electrode surface through the hybridization reaction between the cDNA and aptamer molecules. After each step, the electrode was rinsed with Tris-HCl buffer. Finally, the prepared aptasensor was stored in a refrigerator at 4 °C for further use.

### 2.5. Electrochemical Measurement

The preparation processes of the aptasensor were investigated by cyclic voltammetry (CV) and electrochemical impedance spectroscopy (EIS). CV was performed in the potential range between 0.6 V and −0.1 V at 50 mV/s. EIS was conducted at a potential of 0.23 V in the frequency range of 10^−2^–10^5^ Hz with a sinusoidal voltage amplitude of 5 mV. The electrolyte for CV and EIS tests was 5 mmol/L [Fe(CN)_6_]^3−/4−^ and 0.1 mol/L KCl.

The electrochemical signal of the prepared aptasensor for AFB1 and OTA was recorded by differential pulse voltammetry (DPV). Firstly, 5 μL of Tris-HCl buffer (10 mmol/L, pH = 7.4) containing the analytes was dropped on the prepared aptasensor surface, and then incubated at 37 °C for 50 min. After incubation, the aptasensor was rinsed with Tris-HCl buffer. Finally, DPV test of the aptasensor was performed from −1.2 V to 0.2 V with a quiet time of 200 s using 0.1 mol/L acetic acid buffer solution (pH = 4.5) as electrolyte.

### 2.6. Real Sample Analysis

Three kinds of cereal including corn, wheat, and rice were purchased from a local supermarket (Zhengzhou, China). Samples were extracted with 70% acetonitrile solution, according to the previously reported literature [22]. Firstly, these samples were ground into powder by a cereal grinder. Then, 2 g of each powder sample was mixed with 10 mL of acetonitrile solution (70%, *v*/*v*). Subsequently, the mixture was shaken vigorously for 30 min and centrifuged at 6000 rpm for 10 min. After that, 500 μL of supernatant was transferred to a glass tube, and evaporated to dryness by a stream of nitrogen. Finally, the extracted residue was resolved in 1 mL of Tris-HCl buffer for electrochemical analysis. For recovery test, AFB1 and OTA standard substance were added to the sample powder, and then the contaminated samples were extracted following the above procedure.

### 2.7. Statistical Analysis

T-tests were performed using the IBM SPSS Statistics 27 software package to statistically analyze the aptasensor response for different mycotoxins and the detection results obtained from the aptasensor in real application.

## 3. Results

### 3.1. Principle of the Prepared Electrochemical Aptasensor for AFB1 and OTA Detection

**Figure 1:**
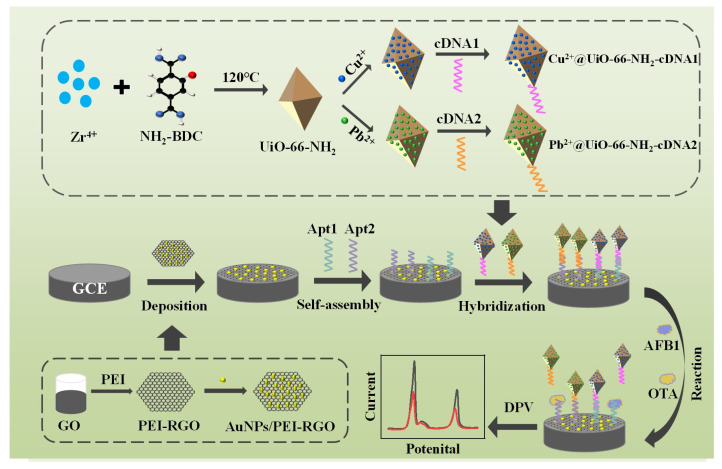
Scheme of the aptasensor preparation and the simultaneous determination of AFB1 and OTA.

The principle of the prepared aptasensor for AFB1 and OTA detection is exhibited in Figure 1. UiO-66-NH_2_ was synthesized by a typical solvothermal method using Zr^4+^ as node and NH_2_-BDC as the linker. After that, UiO-66-NH_2_ absorbed Cu^2+^ and Pb^2+^ and then covalently bonded with cDNA1 and cDNA2, respectively. GO was reduced and compounded with PEI and AuNPs to form AuNPs/PEI-RGO composites. The aptasensor was prepared by the following steps in sequence: deposition of AuNPs/PEI-RGO composites on GCE surface, modification of aptamers by self-assembly, and immobilization of signal probes based on complementary base pairing. Because Cu^2+^ and Pb^2+^ were electroactive, two current peaks were obtained by the prepared aptasensor at different potentials. In the presence of targets, AFB1 and OTA combined with their aptamers, respectively, resulting in the release of signal probes from the sensing interface. Therefore, the obtained electrochemical signals were reduced. The concentrations of AFB1 and OTA were quantified by the changes in peak currents.

### 3.2. Characterization of the Synthesized Materials

**Figure 2:**
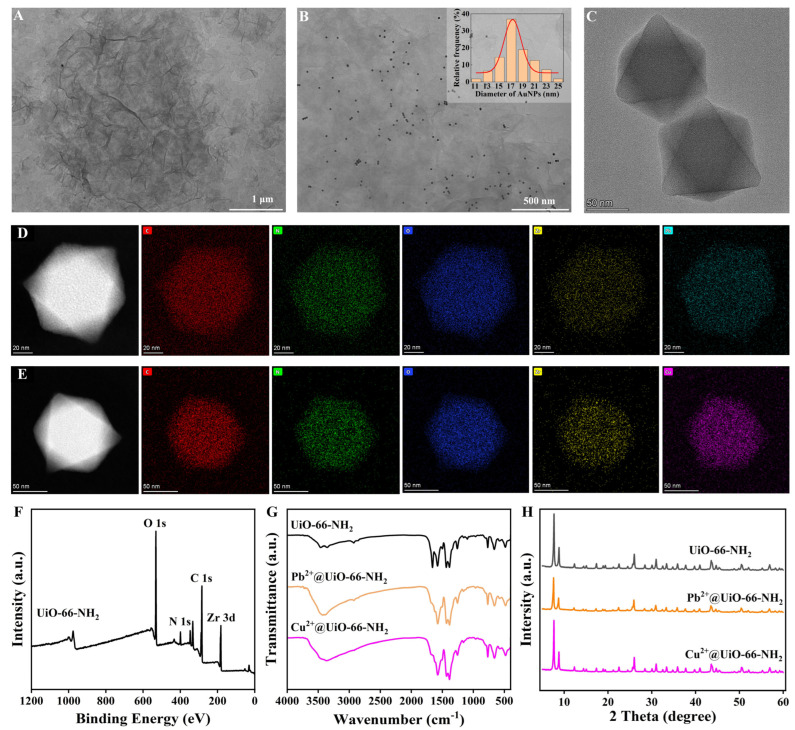
TEM images (**A**) PEI-RGO, (**B**) AuNPs/PEI-RGO, and (**C**) UiO-66-NH_2_. EDS mapping of (**D**) Pb^2+^@UiO-66-NH_2_ and (**E**) Cu^2+^@UiO-66-NH_2_. (**F**) XPS spectrum of UiO-66-NH_2_. (**G**) FT-IR spectra and (**H**) XRD patterns of UiO-66-NH_2_, Pb^2+^@UiO-66-NH_2_, and Cu^2+^@UiO-66-NH_2_.

TEM images of PEI-RGO, AuNPs/PEI-RGO, and UiO-66-NH_2_ are shown in Figure 2A–C. The morphology of PEI-RGO was wrinkled. AuNPs can be observed as dark spots. The diameter of AuNPs was in the range of 11–25 nm with an average diameter of 17 nm. UiO-66-NH_2_ had an octahedral structure. TEM images visually demonstrated the successful preparation of these nanomaterials. As seen in Figure 2D, the EDS mapping images exhibit that the elements of C, N, O, Zr, and Pb were uniformly distributed in Pb^2+^@UiO-66-NH_2_. From Figure 2E, it can be observed that the elements of C, N, O, Zr, and Cu were uniformly distributed in Cu^2+^@UiO-66-NH_2_. The EDS mapping images visually indicate the successful adsorption of Pb^2+^ and Cu^2+^ on UiO-66-NH_2_. The XPS spectrum of UiO-66-NH_2_ is exhibited in Figure 2F. The characteristic element peaks of C 1s, N 1s, O 1s, and Zr 3d are clearly observed in the XPS spectrum. As shown in Figure S1, the peaks at 182.18 eV and 184.68 eV were assigned to Zr 3d_5/2_ and Zr 3d_3/2_ of Zr^4+^ [23]. The XPS spectra of Pb^2+^@UiO-66-NH_2_ and Cu^2+^@UiO-66-NH_2_ are exhibited in the Supplementary Materials. As shown in Figure S2C, the peaks at 143.48 eV and 138.48 eV are due to Pb 4f_5/2_ and Pb 4f_7/2_ of Pb^2+^ [24]. As shown in Figure S3C, the peaks at 952.78 eV and 932.88 eV were ascribed to Cu 2p_1/2_ and Cu 2p_3/2_ of Cu^2+^ [25].

FT-IR spectra of UiO-66-NH_2_, Pb^2+^@UiO-66-NH_2_ and Cu^2+^@UiO-66-NH_2_ are shown in Figure 2G. In the FT-IR spectrum of UiO-66-NH_2_, the bands at 3363 and 3410 cm^−1^ correspond to the symmetric stretching and asymmetric vibrations of N-H bonds; the band at 1574 cm^−1^ was derived from the vibration of C=O bonds; the band at 1496 cm^−1^ was ascribed to the vibration of C-C bonds on benzene; and the band at 1386 cm^−1^ was attributed to the vibration of C-N bonds. Compared with the FT-IR spectrum of UiO-66-NH_2_, the bands at 3363 cm^−1^ and 3410 cm^−1^ in FT-IR spectra of Pb^2+^@UiO-66-NH_2_ and Cu^2+^@UiO-66-NH_2_ were significantly decreased. This could be attributed to metal ions of Pb^2+^ and Cu^2+^ being encapsulated by UiO-66-NH_2_ via the interaction with the amino groups [13].

XRD patterns of UiO-66-NH_2_, Pb^2+^@UiO-66-NH_2_ and Cu^2+^@UiO-66-NH_2_ are shown in Figure 2H. The characteristic diffraction peaks of UiO-66-NH_2_ were similar to the simulated and the reported pattern in the literature [26]. Diffraction peaks at 7.66°, 8.82°, 15.04°, 17.36°, 25.98°, and 30.98°correspond to lattice plane (111), (200), (222), (400), (442), and (711) reflections, respectively. This indicated the octahedral shape of UiO-66-NH_2_ [27]. In addition, these spindly characteristic peaks revealed a high crystallinity. The XRD patterns Pb^2+^@UiO-66-NH_2_ and Cu^2+^@UiO-66-NH_2_ were almost the same as that of UiO-66-NH_2_. This indicated that the crystal structure of UiO-66-NH_2_ was well maintained after the absorption of Pb^2+^ and Cu^2+^.

### 3.3. Electrochemical Characterization

**Figure 3:**
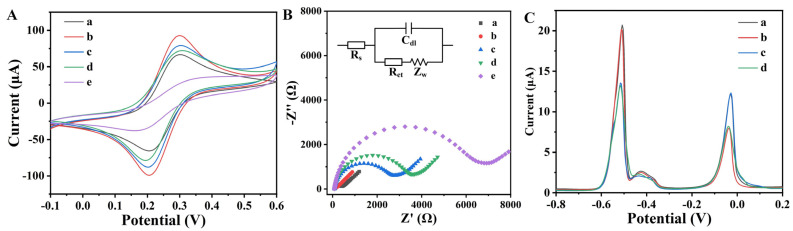
(**A**) CV curves and (**B**) Nyquist plots in 5 mmol/L [Fe(CN)6]^3−/4−^ and 0.1 mol/L KCl of (a) GCE, (b) AuNPs/PEI-RGO/GCE, (c) after the modification of aptamer, (d) after the treatment with MCH, and (e) after the immobilization with Cu^2+^@UiO-66-NH_2_-cDNA1 and Pb^2+^@UiO-66-NH_2_-cDNA2. (**C**) DPV responses of the prepared aptasensor for (a) blank, (b) 10 ng/mL AFB1, (c) 10 ng/mL OTA, and (d) 10 ng/mL AFB1 and OTA.

CV curves of the aptasensor in each preparation step are exhibited in Figure 3A. According to the Randles–Sevcik equation, the peak current on CV curve was positively correlated with the electroactive area of electrochemical sensor. The bare GCE obtained a pair of symmetrical redox peaks. Compared with GCE, the redox peaks obtained by AuNPs/PEI-RGO/GCE significantly increased because the high specific surface area and good electrical conductivity of AuNPs/PEI-RGO composites contributed to enlarging the electroactive area of the electrochemical sensor. In addition, the high specific surface area of AuNPs/PEI-RGO provided more binding sites for aptamer molecules and signal probes, which contributed to improving the sensitivity in detection. After the modification of Apt1 and Apt2 molecules on the electrode surface, the redox peaks decreased because of the poor electrical conductivity of aptamer. After the treatment with MCH, the redox peaks on CV curve further decreased, owing to the blocking effect of MCH. After the immobilization of Cu^2+^@UiO-66-NH_2_-cDNA1 and Pb^2+^@UiO-66-NH_2_-cDNA2, the redox peaks decreased dramatically because UiO-66-NH_2_ and cDNA both exhibited poor electroconductivity. The heterogeneous electron transfer rate (k^0^) was calculated according to the methos developed by Klingler and Kochi [28,29]. The electrode of AuNPs/PEI-RGO/GCE exhibited the fastest transfer kinetics. This demonstrated that the AuNPs/PEI-RGO composites enhanced the current intensity and favored the transfer of charge between the electrode and the solution.

EIS was performed to explore the changes on the electrode/electrolyte interface during the preparation process (Figure 3B). Nyquist plots were fitted by a Randles equivalent circuit. The equivalent circuit included the resistance of the solution (R_s_), the resistance of electron transfer (R_et_), the double-layer capacitance (C_dl_), and the Warburg impedance (Z_w_). In the Nyquist plots, R_s_ was predominant at high frequencies. At intermediate frequencies, R_et_ was expressed as a semicircle, and the diameter of the semicircle corresponded to the R_et_ value. At low frequencies, the straight at low frequencies represented diffusion process [30]. The R_et_ of bare GCE was evaluated to be 332 ± 17 Ω because of the good conductivity of glassy carbon. After the modification of AuNPs/PEI-RGO composites, the R_et_ decreased to 65 ± 5 Ω, owing to the promoted electron transfer. This suggested that AuNPs/PEI-RGO composites were suitable for electroanalytical application owing to their high electrical conductivity. When the thiolated aptamer molecules immobilized on the electrode surface, a large semicircular portion was observed at high frequencies, and the R_et_ significantly increased to 2691 ± 126 Ω. Because the aptamer molecules were electronegative, this hindered the diffusion of [Fe(CN)_6_]^3−/4−^ from the electrolyte to the electrode surface [31]. When MCH was introduced to the electrode surface, the R_et_ further increased to 3536 ± 182 Ω because the nonspecific binding sites were blocked by MCH molecules. When signal probes of Cu^2+^@UiO-66-NH_2_-cDNA1 and Pb^2+^@UiO-66-NH_2_-cDNA2 immobilized on the electrode surface, the R_et_ greatly increased to 6726 ± 329 Ω due to the poor conductivity. This was attributed to the poor conductivity of signal probes that hindered the electron transfer. The above results demonstrated that these nanomaterials and chemicals successively anchored on the electrode surface.

The response signal of the electrochemical aptasensor for AFB1 and OTA was studied by DPV. As shown in Figure 3C, two well-defined peaks at −0.03 V and −0.51 V were obtained in the blank sample. For the blank sample, Cu^2+^@UiO-66-NH_2_-cDNA1 and Pb^2+^@UiO-66-NH_2_-cDNA2 were attached to the electrode surface by complementary base pairing between the aptamer and cDNA. Cu^2+^ and Pb^2+^ as signal producers were reduced to metallic Pb and Cu under low potential and then oxidized during the positive scan of potential, thus generating the current signal. For the sample containing 10 ng/mL AFB1, the peak current at −0.03 V arising from Cu^2+^ decreased to 8.12 μA, while the peak current at −0.51 V did not change significantly. Because AFB1 and cDNA1 competed to bind to Apt1, a number of Cu^2+^@UiO-66-NH_2_-cDNA1 nanoparticles were released from the aptasensor surface. For the sample containing 10 ng/mL OTA, the peak current at −0.51 V arising from Pb^2+^ decreased to 13.23 μA, while the peak current at −0.03 V did not change significantly. Because OTA and cDNA2 competed to bind to Apt2, a number of Pb^2+^@UiO-66-NH_2_-cDNA2 nanoparticles were released from the aptasensor surface. For the sample containing 10 ng/mL of AFB1 and OTA, the peak currents at −0.03 V and −0.51 V decreased simultaneously because the signal probes of Cu^2+^@UiO-66-NH_2_-cDNA1 and Pb^2+^@UiO-66-NH_2_-cDNA2 were released from the aptasensor surface concurrently due to the existence of AFB1 and OTA. The change in peak current at −0.03 V arising from Cu^2+^ was correlative with the AFB1 concentration. The change in peak current at −0.51 V arising from Pb^2+^ was correlative with the OTA concentration. These results indicated that the prepared aptasensor could detect AFB1 and OTA simultaneously.

### 3.4. Optimization of the Aptasensor Performance

**Figure 4:**
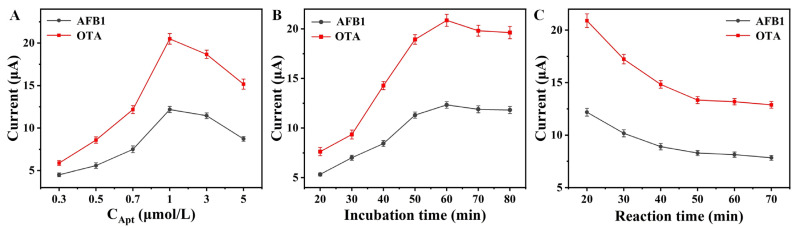
Effects of (**A**) aptamer concentration, (**B**) the incubation time for signal probes, and (**C**) the reaction time for AFB1 and OTA on the current signal (n = 3).

To improve the performance of the aptasensor, some key conditions were optimized, including aptamer concentration, the incubation time for signal probes, and the reaction time for AFB1 and OTA. From Figure 4A, the peak currents increased with the aptamer concentration from 0.3 to 1 μmol/L, and then decreased slightly with a higher concentration. This is probably because the excess aptamer molecules inhibited the electron transfer. From Figure 4B, the peak currents increased with the incubation time for signal probes and became stable after 60 min because the immobilized signal probes nearly reached saturation on the aptasensor surface. From Figure 4C, the peak currents decreased with the reaction time for AFB1 and OTA from 20 to 50 min, and then leveled off. This might be because the reaction between aptamers and targets reached equilibration. These optimal conditions (1 μmol/L of aptamer concentration, 60 min of incubation time, and 50 min of reaction time) were used for the subsequent experiments.

### 3.5. Analytical Performance of the Aptasensor

**Figure 5:**
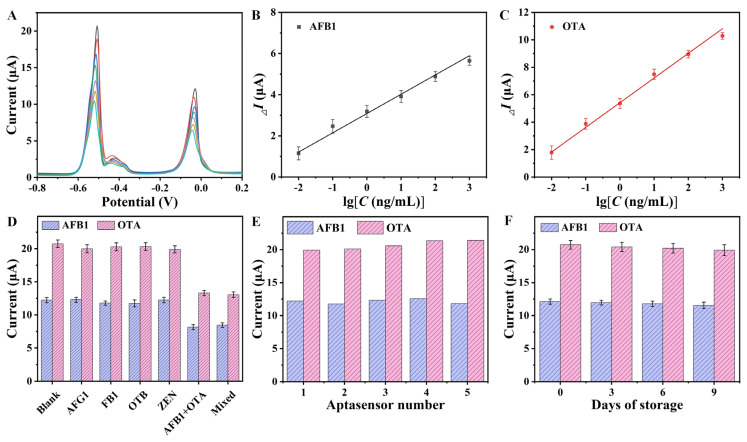
(**A**) DPV curves for AFB1 and OTA with different concentrations (0, 0.01, 0.1, 1, 10, 100, 1000 ng/mL). The calibration plots of *_△_I* vs. the logarithm of (**B**) AFB1 and (**C**) OTA concentrations. (**D**) Current signals of the aptasensor for 10 ng/mL different mycotoxins; (**E**) current signals of the different aptasensors; (**F**) current signals of the aptasensor for different storage days (n = 3).

The analytical performance of the aptasensor was evaluated to detect different concentrations of AFB1 and OTA under optimal conditions. As shown in Figure 5A, the peak currents at −0.03 V and −0.51 V decreased as AFB1 and OTA concentration increased. The calibration plots of *_△_I* vs. the logarithm of AFB1 and OTA concentration displayed a good linear relationship in a dynamic range from 0.01 to 1000 ng/mL (Figure 5B,C). The linear equations were established as *_△_I* = 3.08 + 0.94 × lg*C* for AFB1 and *_△_I* = 5.42 + 1.79 × lg*C* for OTA. The limit of detection (LOD) was calculated according to the following equation: LOD = 3δ/S; δ was the standard deviation of the signal responses for blank samples; S was the slope of the standard curve [32]. The LOD was estimated to be 6.2 ng/L for AFB1 and 3.7 ng/L for OTA. Compared with previously reported aptasensors (Table 1), the proposed aptasensor exhibited better performance in terms of LOD and linear range. It was attributed to the combined effects from AuNPs/PEI-RGO composites and the prepared signal probes. The modified AuNPs/PEI-RGO composites on GCE provided a large electroactive area. Accordingly, plenty of aptamer molecules were immobilized on the sensing interface. In addition, the signal probes containing abundant Pb^2+^ and Cd^2+^ produced large current signals, resulting in high sensitivity.

The selectivity of the aptasensor was investigated by the analysis of other commonly found mycotoxins, including AFG1, FB1, OTB, and ZEN. The concentration of these interferents was 10 ng/mL, which was within the typical concentration ranges for these substances. Figure 5D exhibits the peak currents by the aptasensor for 10 ng/mL of these mycotoxins. A *t*-test was performed using the IBM SPSS Statistics 27 software package for an analysis of significance. The peak currents by the aptasensor significantly decreased in the analysis of AFB1 and OTA (*p* < 0.05), while the other species did not exhibit a significant difference in peak currents (*p* > 0.05). In the presence of AFB1 and OTA, they specifically combined with their aptamers, respectively, resulting in the release of signal probes from the sensing interface. Therefore, the obtained current peaks decreased. The proposed aptasensor exhibited an excellent selectivity towards AFB1 and OTA, which mainly depended on the specific recognition of the aptamer. To estimate the reproducibility, a batch of aptasensors were freshly prepared and the current signals were collected (Figure 5E). The RSD value of the peak currents from the five aptasensors was 3.34% for AFB1 and 2.86% for OTA, indicating good reproducibility. To evaluate the stability, the aptasensors were stored at 4 °C for a different number days (Figure 5F). The current signals still reached above 95% of the initial currents, indicating an acceptable stability.

### 3.6. Determination of AFB1 and OTA in Cereal Samples

Cereal samples including corn, wheat, and rice were analyzed to evaluate the feasibility of the proposed aptasensor because none of AFB1 and OTA residual was found in the original samples either by HPLC-MS or the proposed aptasensor. These cereal samples were spiked with different amounts of AFB1 and OTA for analysis. From Table 2, recovery rates of the spiked samples were in the range of 92.5–104.1%. From Table S4, *p* values from *t*-test were greater than 0.05, suggesting that there was no significant difference between the spiked content and the detected result provided by the aptasensor. In addition, these samples were analyzed by HPLC-MS to verify the accuracy of the proposed aptasensor. The details of the HPLC-MS procedure are provided in the Supplementary Materials. An HPLC-MS chromatogram is exhibited in Figure S4. AFB1 and OTA exhibited good separation at different retention times in the chromatogram. The concentrations of AFB1 and OTA from both the proposed aptasensor and HPLC-MS were in good agreement with each other (Table 2), indicating a good accuracy. The above results demonstrated that the proposed aptasensor had a satisfactory level of reliability in a real application scenario.

## 4. Conclusions

A novel electrochemical aptasensor was developed for the simultaneous determination of AFB1 and OTA. AuNPs/PEI-RGO composites were used as a sensing substrate for the immobilization of aptamers. UiO-66-NH_2_ loaded with metal ions of Cu^2+^ and Pb^2+^ served as signal producers for the simultaneous measurement of AFB1 and OTA, respectively. Thanks to the highly conductive sensing substrate and the favorable signal producers, the prepared aptasensor exhibited high sensitivity, a low detection limit, and a wide linear detection range. In the application of cereal samples, good accuracy and satisfactory recovery were demonstrated by the prepared aptasensor. Benefiting from these advantageous features, the prepared aptasensor offers great prospects for the simultaneous determination of AFB1 and OTA in cereals.

## Figures and Tables

**Table 1:** The performance levels of the prepared aptasensor and other electrochemical aptasensors from the literature.

Electrochemical Sensor	**Analytes**	Linear Ranges (ng/mL)	LOD (ng/mL)	Reference
Aptamer/Fe_3_O_4_@Au/SPCE	AFB1	0.02–50	0.015	[33]
Fc-aptamer/cDNA/AuNPs/THI-rGO/GCE	AFB1	0.05–20	0.016	[34]
Ab/Au-PANI/ITO	AFB1	0.1–100	0.05	[35]
Aptamer/diazonium/SPCE	OTA	0.15–2.5	0.15	[36]
Cd-MOF@Au/aptamer/cDNA/AuNPs-MoS_2_/GCE	OTA	0.05–100	0.01	[37]
Apt/FcL/AuNPs/GCE	OTA	0.5–70	0.011	[38]
The prepared aptasensor	AFB1 OTA	0.01–1000	0.0062 0.0037	This work

SPCE: screen-printed carbon electrode; Fc: ferrocene; THI: thionine; Ab: antibody; PANI: polyaniline; FcL: ferrocenyl lipoic acid ester.

**Table 2:** Analysis of AFB1 and OTA content in cereal samples (n = 3).

Sample	Spiked (ng/g)		Detected by the Aptasensor (ng/g)		Recovery		Detected by HPLC-MS (ng/g)	
	AFB1	OTA	AFB1	OTA	AFB1	OTA	AFB1	OTA
Corn	1	1	1.04 ± 0.05	0.97 ± 0.04	103.7%	96.9%	1.02 ± 0.05	1.05 ± 0.04
	50	50	52.06 ± 2.25	46.94 ± 1.90	104.1%	93.9%	47.10 ± 1.78	51.12 ± 2.23
Wheat	5	5	4.72 ± 0.18	5.16 ± 0.24	94.4%	103.2%	5.22 ± 0.23	4.79 ± 0.19
	100	100	93.90 ± 3.41	95.01 ± 4.21	93.9%	95.0%	91.06 ± 3.90	94.35 ± 4.15
Rice	25	25	24.34 ± 1.17	25.59 ± 0.95	97.4%	102.4%	25.07 ± 1.05	24.80 ± 0.92
	500	500	462.53 ± 18.72	472.96 ± 21.07	92.5%	94.6%	483.57 ± 16.35	467.32 ± 19.16

## Data Availability

The original contributions presented in the study are included in the article/Supplementary Materials, and further inquiries can be directed to the corresponding authors.

## Supplementary Materials

The following supporting information can be downloaded at: https://www.mdpi.com/article/10.3390/foods13142177/s1, Table S1. The procedure of mobile phase gradient elution. Table S2. Mass spectrometry conditions for HPLC-MS detection. Table S3. The values of _△_Ep, I_pa_/I_pc_, and K^0^. Table S4. T-test of the proposed aptasensor method. Figure S1. XPS spectrum of UiO-66-NH_2_ with high-resolution of Zr 3d. Figure S2. (A) XPS spectrum of Pb^2+^@UiO-66-NH_2_, and high-resolution of (B) Zr 3d, (C) Pb 4f, (D) O 1s, (E)N 1s, (F) C 1s. Figure S3. (A) XPS spectrum of Cu^2+^@UiO-66-NH_2_, and high-resolution of (B) Zr 3d, (C) Cu 2p, (D) O 1s, (E)N 1s, (F) C 1s. Figure S4. The HPLC-MS chromatogram for ABF1 and OTA.

## References

[1] Campagnollo F.B., Ganev K.C., Khaneghah A.M., Portela J.B., Cruz A.G., Granato D., Corassin C.H., Oliveira C.A.F., Sant’Ana A.S. (2016). The occurrence and effect of unit operations for dairy products processing on the fate of aflatoxin M1: A review. Food Control.

[2] Milani J., Maleki G. (2014). Effects of processing on mycotoxin stability in cereals. J. Sci. Food Agric..

[3] Joubrane K., Mnayer D., El Khoury A., El Khoury A., Awad E. (2020). Co-Occurrence of Aflatoxin B1 and Ochratoxin A in Lebanese Stored Wheat. J. Food Prot..

[4] De Girolamo A., Lippolis V., Pascale M. (2022). Overview of Recent Liquid Chromatography Mass Spectrometry-Based Methods for Natural Toxins Detection in Food Products. Toxins.

[5] Ong J.Y., Pike A., Tan L.L. (2021). Recent Advances in Conventional Methods and Electrochemical Aptasensors for Mycotoxin Detection. Foods.

[6] Oplatowska-Stachowiak M., Sajic N., Xu Y., Haughey S.A., Mooney M.H., Gong Y.Y., Verheijen R., Elliott C.T. (2016). Fast and sensitive aflatoxin B1 and total aflatoxins ELISAs for analysis of peanuts, maize and feed ingredients. Food Control.

[7] Wu L., Zhou M., Wang Y., Liu J. (2020). Nanozyme and aptamer- based immunosorbent assay for aflatoxin B1. J. Hazard. Mater..

[8] Wu Q., Tao H., Wu Y., Wang X., Shi Q., Xiang D. (2022). A Label-Free Electrochemical Aptasensor Based on Zn/Fe Bimetallic MOF Derived Nanoporous Carbon for Ultra-Sensitive and Selective Determination of Paraquat in Vegetables. Foods.

[9] Liu X., Zhang X. (2015). Aptamer-Based Technology for Food Analysis. Appl. Biochem. Biotechnol..

[10] Nan M.-N., Bi Y., Xue H.-L., Long H.-T., Xue S.-L., Pu L.-M., Prusky D. (2021). Modification performance and electrochemical characteristics of different groups of modified aptamers applied for label-free electrochemical impedimetric sensors. Food Chem..

[11] Xiong Y., Li W., Wen Q., Xu D., Ren J., Lin Q. (2022). Aptamer-engineered nanomaterials to aid in mycotoxin determination. Food Control.

[12] Feng L.-N., Bian Z.-P., Peng J., Jiang F., Yang G.-H., Zhu Y.-D., Yang D., Jiang L.-P., Zhu J.-J. (2012). Ultrasensitive Multianalyte Electrochemical Immunoassay Based on Metal Ion Functionalized Titanium Phosphate Nanospheres. Anal. Chem..

[13] Chen M., Gan N., Zhou Y., Li T., Xu Q., Cao Y., Chen Y. (2016). An electrochemical aptasensor for multiplex antibiotics detection based on metal ions doped nanoscale MOFs as signal tracers and RecJf exonuclease-assisted targets recycling amplification. Talanta.

[14] Chen M., Gan N., Zhou Y., Li T., Xu Q., Cao Y., Chen Y. (2017). A novel aptamer- metal ions- nanoscale MOF based electrochemical biocodes for multiple antibiotics detection and signal amplification. Sens. Actuators B Chem..

[15] Wang J.-C., Hu Y.-H., Chen G.-J., Dong Y.-B. (2016). Cu(ii)/Cu(0)@UiO-66-NH2: Base metal@MOFs as heterogeneous catalysts for olefin oxidation and reduction. Chem. Commun..

[16] Xu Y., Zhang W., Shi J., Zou X., Li Z., Zhu Y. (2016). Microfabricated interdigitated Au electrode for voltammetric determination of lead and cadmium in Chinese mitten crab (*Eriocheir sinensis*). Food Chem..

[17] Yiwei X., Wen Z., Xiaowei H., Jiyong S., Xiaobo Z., Yanxiao L., Xueping C., Tahir H.E., Zhihua L. (2018). A Self-assembled L-Cysteine and Electrodeposited Gold Nanoparticles-reduced Graphene Oxide Modified Electrode for Adsorptive Stripping Determination of Copper. Electroanalysis.

[18] Ruscito A., Smith M., Goudreau D.N., DeRosa M.C. (2016). Current Status and Future Prospects for Aptamer-Based Mycotoxin Detection. J. AOAC Int..

[19] Kandiah M., Nilsen M.H., Usseglio S., Jakobsen S., Olsbye U., Tilset M., Larabi C., Quadrelli E.A., Bonino F., Lillerud K.P. (2010). Synthesis and Stability of Tagged UiO-66 Zr-MOFs. Chem. Mater..

[20] Qiao W., He B., Ren W., Zhao R., Suo Z., Yan H., Xu Y., Wei M., Jin H. (2022). Colloidal Au sphere and nanoflower-based immunochromatographic strips for sensitive detection of zearalenone in cereals. Anal. Methods.

[21] Wang B., He B., Xie L., Cao X., Liang Z., Wei M., Jin H., Ren W., Suo Z., Xu Y. (2022). A novel detection strategy for nitrofuran metabolite residues: Dual-mode competitive-type electrochemical immunosensor based on polyethyleneimine reduced graphene oxide/gold nanorods nanocomposite and silica-based multifunctional immunoprobe. Sci. Total Environ..

[22] Guan X., Feng Y., Suo D., Xiao Z., Wang S., Liang Y., Fan X. (2022). Simultaneous Determination of 11 Mycotoxins in Maize via Multiple-Impurity Adsorption Combined with Liquid Chromatography–Tandem Mass Spectrometry. Foods.

[23] Peñas-Garzón M., Sampaio M.J., Wang Y.L., Bedia J., Rodriguez J.J., Belver C., Silva C.G., Faria J.L. (2022). Solar photocatalytic degradation of parabens using UiO-66-NH_2_. Sep. Purif. Technol..

[24] Wang C., Xiong C., He Y., Yang C., Li X., Zheng J., Wang S. (2021). Facile preparation of magnetic Zr-MOF for adsorption of Pb(II) and Cr(VI) from water: Adsorption characteristics and mechanisms. Chem. Eng. J..

[25] Zhang Y., Yuan K., Magagnin L., Wu X., Jiang Z., Wang W. (2023). Selective adsorption of Pb (II) and Cu (II) on mercapto-functionalized aerogels: Experiments, DFT studies and LCA analysis. J. Clean. Prod..

[26] Wang X., Xu Y., Li Y., Li Y., Li Z., Zhang W., Zou X., Shi J., Huang X., Liu C. (2021). Rapid detection of cadmium ions in meat by a multi-walled carbon nanotubes enhanced metal-organic framework modified electrochemical sensor. Food Chem..

[27] Xu Y., Li X., Zhang W., Jiang H., Pu Y., Cao J., Jiang W. (2021). Zirconium(IV)-based metal-organic framework for determination of imidacloprid and thiamethoxam pesticides from fruits by UPLC-MS/MS. Food Chem..

[28] Klingler R.J., Kochi J.K. (1981). Electron-transfer kinetics from cyclic voltammetry. Quantitative description of electrochemical reversibility. J. Phys. Chem..

[29] Zamarchi F., Silva T.R., Winiarski J.P., Santana E.R., Vieira I.C. (2022). Polyethylenimine-Based Electrochemical Sensor for the Determination of Caffeic Acid in Aromatic Herbs. Chemosensors.

[30] da Silva V.J., Baumgarten L.G., Dreyer J.P., Santana E.R., Spinelli A., Winiarski J.P., Vieira I.C. (2024). Heparin-stabilized gold nanoparticles embedded in graphene for the electrochemical determination of esculetin. Anal. Methods.

[31] Dong S., Zhao R., Zhu J., Lu X., Li Y., Qiu S., Jia L., Jiao X., Song S., Fan C. (2015). Electrochemical DNA Biosensor Based on a Tetrahedral Nanostructure Probe for the Detection of Avian Influenza A (H7N9) Virus. ACS Appl. Mater. Interfaces.

[32] Pu X., Hu Y., Niu M., Liu H., Li C., Ma W., Gu Y. (2024). An antifouling electrochemical aptasensor based on a Y-shaped peptide for tetracycline detection in milk. J. Food Compos. Anal..

[33] Wang C., Qian J., An K., Ren C., Lu X., Hao N., Liu Q., Li H., Huang X., Wang K. (2018). Fabrication of magnetically assembled aptasensing device for label-free determination of aflatoxin B1 based on EIS. Biosens. Bioelectron..

[34] Li Y., Liu D., Zhu C., Shen X., Liu Y., You T. (2020). Sensitivity programmable ratiometric electrochemical aptasensor based on signal engineering for the detection of aflatoxin B1 in peanut. J. Hazard. Mater..

[35] Yagati A.K., Chavan S.G., Baek C., Lee M.-H., Min J. (2018). Label-Free Impedance Sensing of Aflatoxin B1 with Polyaniline Nanofibers/Au Nanoparticle Electrode Array. Sensors.

[36] Mishra R.K., Hayat A., Catanante G., Ocaña C., Marty J.-L. (2015). A label free aptasensor for Ochratoxin A detection in cocoa beans: An application to chocolate industries. Anal. Chim. Acta.

[37] Li D.-l., Zhang X., Ma Y., Deng Y., Hu R., Yang Y. (2018). Preparation of an OTA aptasensor based on a metal–organic framework. Anal. Methods.

[38] Argoubi W., Algethami F.K., Raouafi N. (2024). Enhanced sensitivity in electrochemical detection of ochratoxin A within food samples using ferrocene- and aptamer-tethered gold nanoparticles on disposable electrodes. RSC Adv..

