# Supplementary material for: Simultaneous Determination of Aflatoxin B1 and Ochratoxin A in Cereals by a Novel Electrochemical Aptasensor Using Metal–Organic Framework as Signal Carrier
Source: Foods. 2024 Jul 10;13(14):2177. doi: 10.3390/foods13142177 (PMC11276064; doi:10.3390/foods13142177)
Supplement: Supplementary file 1 [file foods-13-02177-s001.zip › foods-2988919-supplementary.pdf]

Supplementary Material

Simultaneous Determination of Aflatoxin B1 and Ochratoxin A in Cereals by a Novel Electrochemical Aptasensor Using Metal–Organic Framework as Signal Carrier

Yiwei Xu [1,*], Xupeng Jia [1], Sennan Yang [2], Mengrui Cao [1], Baoshan He [1,*], Wenjie Ren [1] and Zhiguang Suo [1]

[1] School of Food Science and Technology, National Engineering Research Center of Wheat and Corn Further Processing, Henan University of Technology, Zhengzhou 450001, China; jiaxupeng2000@163.com (X.J.); caomengrui1213@126.com (M.C.); wjren0818@163.com (W.R.); zg_suo@163.com (Z.S.)

[2] Henan Institute of Food and Salt Industry Inspection Technology, Zhengzhou 450003, China; 18595505932@163.com

* Correspondence: xu_yiwei@126.com (Y.X.); baoshanhe2008@haut.edu.cn (B.H.)

HPLC-MS conductions

The chromatographic column was a Waters BEH C18 column (100 mm×3.0 mm, 1.7 μm). The column temperature was 40 °C. The injection volume was 10 μL. The mobile phase flow rate was 0.3 mL/min. The mobile phase compositions and elution gradients was exhibited in Table S1.

Table S1 The procedure of mobile phase gradient elution.

Time (min)	0.1% Formic Acid (%)	Acetonitrile (%)
0.0	80	20
0.5	80	20
2.3	40	60
4.0	40	60
5.0	0	100
6.0	0	100
6.5	80	20
8.0	80	20

Mass spectrometry was performed using an electrospray ionization source (positive ion switching) in multi-reaction monitoring mode. The spray voltage was 3 kV. The ion source temperature was 300 °C. The collision gas was nitrogen. The specific parameters were shown in Table S2.

Table S2 Mass spectrometry conditions for HPLC-MS detection.

Toxin	Precursor Ion (M/Z)	Fragment Ion (M/Z)	Cone Voltage (V)	Collision Energy (V)
AFB1	313.0	241.1	25	51
		285.0	25	30
OTA	404.0	239.0	45	30
		358.0	45	26

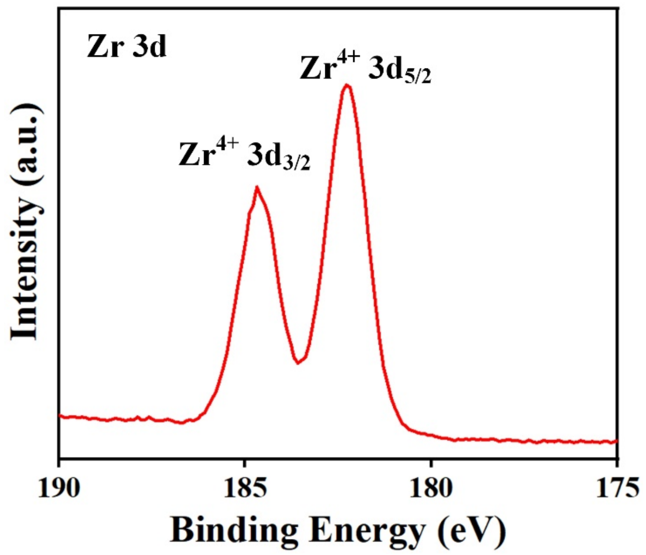

Figure S1. XPS spectrum of UiO-66-NH_2 with high-resolution of Zr 3d.

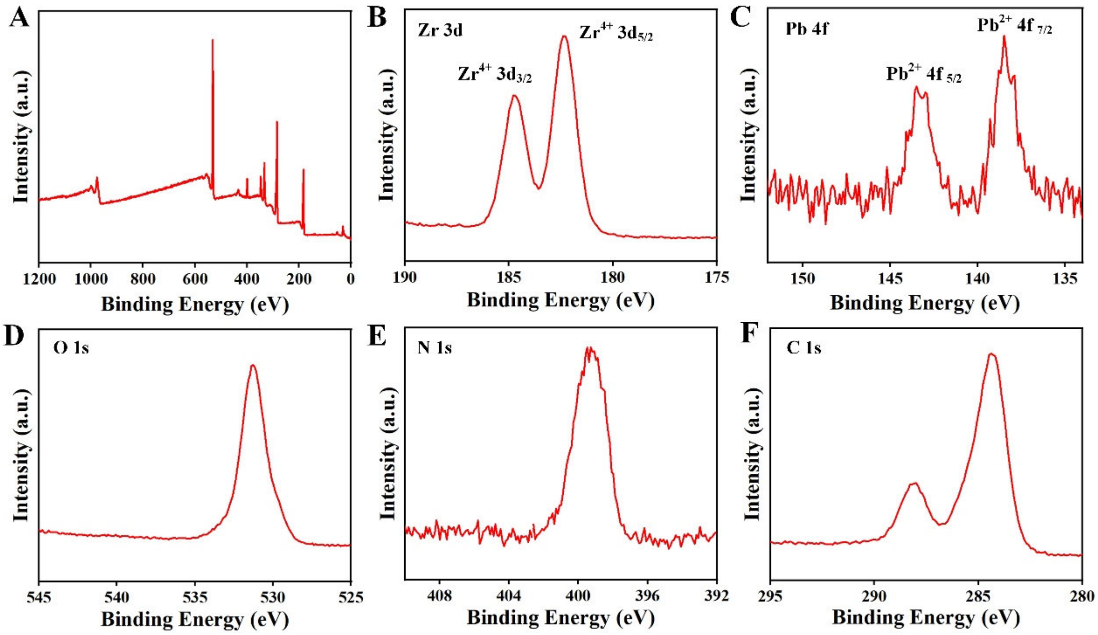

Figure S2. (A) XPS spectrum of Pb^{2+}@UiO-66-NH_2, and high-resolution of (B) Zr 3d, (C) Pb 4f, (D) O 1s, (E)N 1s, (F) C 1s.

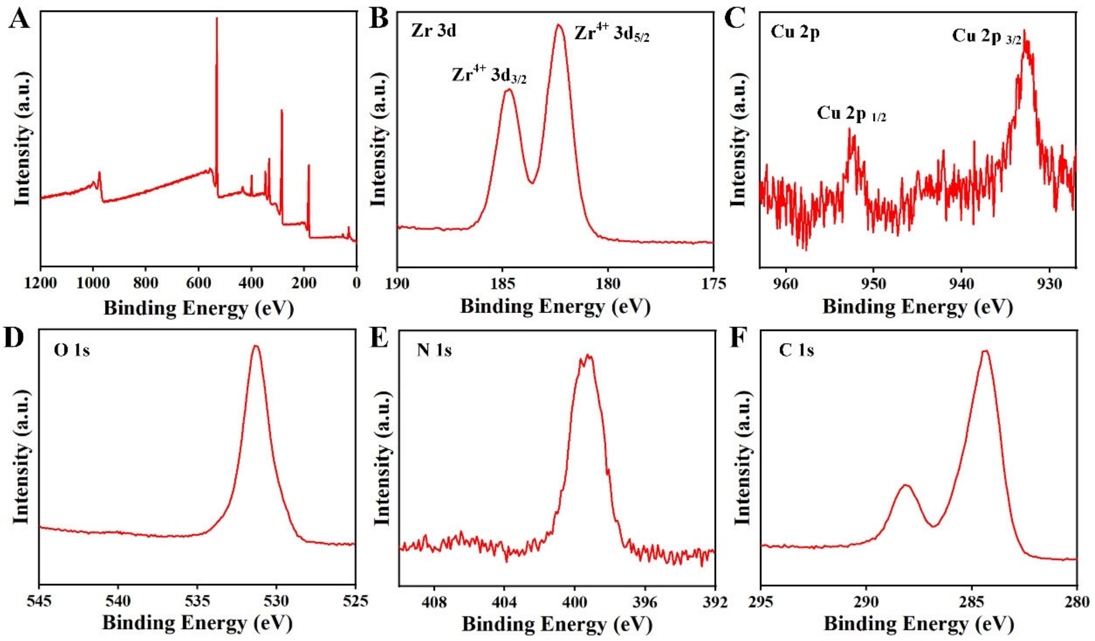

Figure S3. (A) XPS spectrum of Cu^{2+}@UiO-66-NH_2, and high-resolution of (B) Zr 3d, (C) Cu 2p, (D) O 1s, (E)N 1s, (F) C 1s.

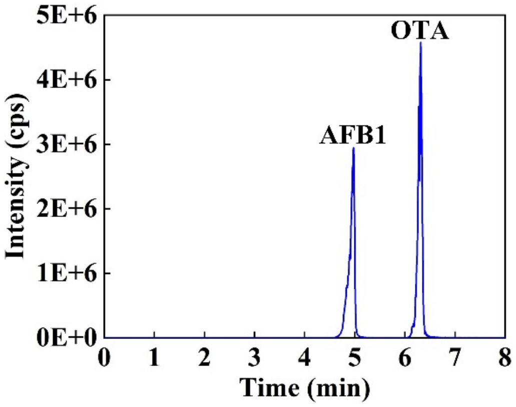

Figure S4. The HPLC-MS chromatogram for ABF1 and OTA.

The heterogeneous electron transfer rate (k^0) in each electrode was calculated according to the methos developed by Klingler and Kochi in Equation (S1)

$$k^0 = 2.18(\frac{\alpha DnvF}{RT})^{\frac{1}{2}} e^{-(\frac{\alpha^2 nF}{RT})\Delta E} \quad \text{(S1)}$$

where α is the transfer coefficient (α=0.5), n the number of electrons transferred in the redox reaction (n=1), F is the Faraday constant (96485 C mol^{-1}), D refers to the diffusion coefficient of the $K_4[Fe(CN)_6]$ species in aqueous solution (6.7×10^{-6} cm^2 s^{-1}), v is the potential scan rate (V s^{-1}), R is the universal gas constant (8.314 J K^{-1} mol^{-1}), and T is the absolute temperature.

Table S3 The values of △Ep, I_{pa}/I_{pc}, and K^0.

Modification step	△Ep (V)	I_{pa}/I_{pc}	K^0 (cm s^{-1})
a	0.097	1.02	3.06
b	0.095	0.95	3.12
c	0.101	0.91	2.95
d	0.114	0.93	2.60
e	0.157	0.85	1.71

a: GCE; b: AuNPs/PEI-RGO/GCE; c: after the modification of aptamer; d: after the treatment with MCH; e: after the immobilization with Cu^{2+}@UiO-66-NH_2-cDNA1 and Pb^{2+}@UiO-66-NH_2-cDNA2.

Table S4 T-test of the proposed aptasensor method.

Sample	Mycotoxin	Spiked (ng/g)	Detected by the aptasensor (ng/g)	T value	P value
Corn	AFB1	1	1.04±0.05	1.155	0.368
		50	52.06±2.25	1.293	0.325
	OTA	1	0.97±0.04	-1.039	0.408
		50	46.94±1.90	-2.275	0.151
Wheat	AFB1	5	4.72±0.18	-2.204	0.158
		100	93.90±3.41	-2.516	0.128
	OTA	5	5.16±0.24	0.924	0.453
		100	95.01±4.21	-1.675	0.236
Rice	AFB1	25	24.34±1.17	-0.799	0.508
		500	462.53±18.72	-2.832	0.105
	OTA	25	25.59±0.95	0.881	0.471
		500	472.96±21.07	-1.815	0.211
